# Building an agile state-wide research infrastructure to address COVID-19 and emerging threats: insights from an equity-centered public health and academic collaboration in California

**DOI:** 10.3389/fpubh.2025.1549326

**Published:** 2025-07-08

**Authors:** Priya B. Shete, Nicole Santos, Hilary Spindler, Tomás León, Maya Petersen, A. Marm Kilpatrick, Seema Jain, James Watt, Rohan Radhakrishna, Erica Pan, Tomás Aragón

**Affiliations:** ^1^Department of Pulmonary and Critical Care, University of California San Francisco, San Francisco, CA, United States; ^2^Department of Epidemiology and Biostatistics, University of California San Francisco, San Francisco, CA, United States; ^3^California Department of Public Health, Sacramento, CA, United States; ^4^Department of Epidemiology and Biostatistics, University of California Berkeley, Berkeley, CA, United States; ^5^Department of Ecology and Evolutionary Biology, University of California Santa Cruz, Santa Cruz, CA, United States

**Keywords:** data sharing, evidence to policy translation, research prioritization, modeling, collaboration

## Abstract

The COVID-19 pandemic underscored the need for efficient real-time evidence generation to inform public health interventions and policies. To address this gap, a formalized research partnership between the California Department of Public Health (CDPH) and the University of California (UC) was created. The aim of this case study is to describe the achievements and lessons learned from the California Collaborative for Public Health Research (CPR3). This state-wide infrastructure (1) streamlines data sharing and use between UC researchers and public health agencies; (2) sets priority research agendas that reflect the needs of the state’s diverse communities; and (3) fosters research collaboration and evidence translation. This partnership may serve as a guide for how academic and public health entities can jointly prioritize, conduct, and act upon policy-relevant research for current and emerging threats.

## Introduction

In California, COVID-19 has claimed over 100,000 lives with notable racial disparities in hospitalization and death rates that changed throughout the pandemic (1). COVID-19 has further impacted individuals, families and communities in ways that have not yet been fully realized or understood. For example, losses in learning, jobs, and housing, combined with changes in social interactions and access to services and support, have significantly affected physical and mental health and well-being of our communities (2). Pandemic mitigation strategies reduced transmission and saved lives (3–5); yet some, such as remote school instruction and telework, increased social disconnection and mental distress among both youth and adults (6, 7). Existing social and economic disparities were exacerbated among already marginalized individuals and communities, including those who had pre-existing conditions or disabilities, relied on social services, lived in rural or underserved settings, and/or had ongoing unmet needs related to stable income, housing, or food security (8, 9).

In the face of these challenges and the unprecedented complexity inherent to the pandemic, the spread of COVID-19 presented a pivotal moment for public health. Transparent, data-informed decision-making was critical (10). While academic researchers were available to conduct high-quality research to supplement public health evidence generation, the systems needed to enable collaboration between academia and public health agencies were inefficient or non-existent in many cases (11). Most academic institutions did not have access to data at public health agencies to conduct rapid, population-level, policy-focused public health research. Direct engagement between decision-makers and academic researchers was often based on pre-existing relationships, making the bi-directional sharing of relevant data, evidence, priorities, and resources inconsistent and insufficient. Furthermore, the dissemination of research using traditional peer-reviewed publication processes was often delayed, mitigating the use of relevant findings, contributing to the increase in non-peer reviewed studies and contrasting views in the scientific literature and media landscape (12).

The pandemic also revealed the potential for how community-academic-government-partnerships could be the cornerstone of many local public health responses (13–15). Uptake of interventions and adherence to mitigation strategies often depended on public support and trust (16–19), highlighting the critical role community partners play in intervention prioritization and implementation. The pandemic reiterated the need for tailored strategies to support community-engaged public health decision-making and also emphasized the need for agile models to foster multi-disciplinary research collaboration that incorporates community perspectives.

With the intent of identifying opportunities to enhance public health research in future responses, we describe a successful academic-public health research partnership that was established in the midst of the COVID-19 pandemic between the University of California (UC) and the California Department of Public Health (CDPH). Initially launched with an emphasis on modeling and advanced analytics in early 2021, this state-wide partnership identified priority pandemic topics as they emerged and catalyzed the rapid generation of real-time evidence which directly informed public health recommendations and policies, such as those related to stay-at-home orders, vaccine allocation, school and business re-opening (20–23). The partnership has since evolved from focusing on the acute phase of COVID-19 to long-term, equitable recovery and resilience that integrates community perspectives in research prioritization and implementation. Named the California Collaborative for Public Health Research (CPR3), this type of collaborative infrastructure can extend beyond the pandemic to other public health priorities, such as behavioral health, emerging pathogens, vector borne diseases, and health impacts due to climate change. The objective of this paper is to describe the insights and lessons learned from this successful program.

## Context

This UC-CDPH partnership directly links multi-disciplinary academics, who bring methodological and analytical expertise for evidence generation, and public health departments, who are responsible for collection of disease surveillance data, disease control implementation, allocation of resources, and policymaking.

The UC system is a leading state-based public research university system that comprises 10 campuses. It is operated by 227,000 faculty and staff and includes over 170 academic disciplines (24). Thus, the UC system is uniquely positioned to contribute to an equitable pandemic recovery and the overall public health goals of the state. Moreover, half of the campuses are academic health centers (collectively known as UC Health), providing primary and specialist health services to many Californians and training the next generation of service providers. Support from the UC Office of the President (UCOP), UC Vice Chancellors of Research, and the UC Research and Innovation Office further positions the UC system to address the needs of the diverse communities across the state.

The CDPH is a state health department whose mission is “to advance the health and well-being of California’s diverse people and communities” (25). As a subdivision of the California Health and Human Services Agency (CalHHS) supporting 61 local health jurisdictions, CDPH offers comprehensive services spanning nutrition, infectious disease, health promotion, chronic disease, and behavioral health. It implements programs, provides services, and conducts program evaluation and disease surveillance in collaboration with local, state, and federal partners for over 39 million Californians.

## Programmatic elements

**Figure 1:**
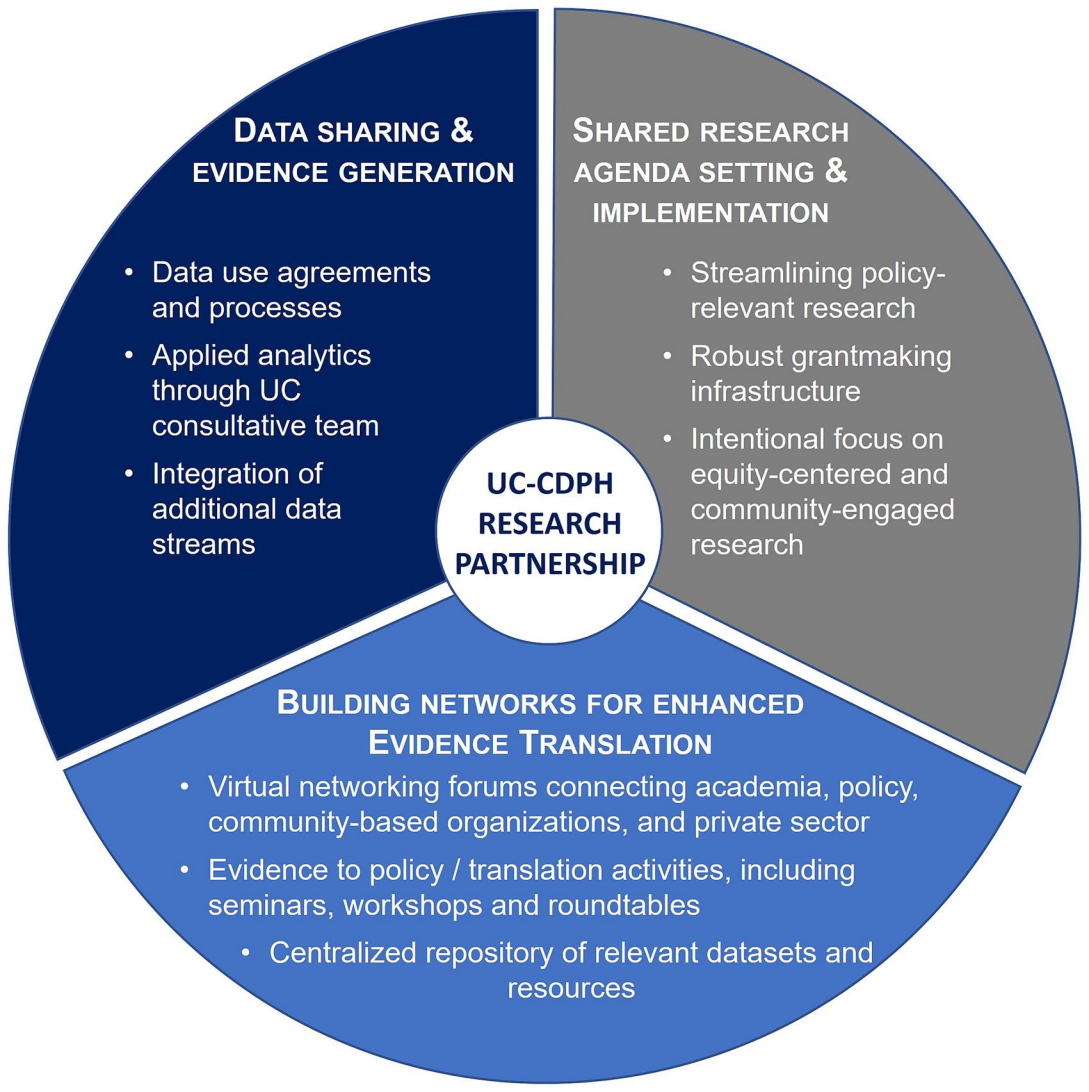
Three components of the California Collaborative for Public Health Research, a state-wide academic-public health partnership between University of California (UC) and California Department of Public Health (CDPH).

Three components have been central to this state-wide academic-public health research partnership (Figure 1): (1) data sharing and evidence generation, (2) shared research agenda setting and implementation, and (3) building networks for enhanced evidence translation. Multi-stakeholder governance structures, which have evolved as research priorities transitioned over time, provide critical guidance on key activities and vision setting for each of these components (Table 1). Inclusion of various perspectives – from public health, academia, community-based organizations, state agencies, and private sector – has been critical to the success of this partnership. We are advised by leaders of community-based organizations who provide diverse perspectives on health and public health priorities across the state.

**Table 1:** CPR3 governance and advisory structures.

Governance committee	Representatives	Areas of oversight
Executive Steering Committee	CDPH/CalHHS leadership Multi-disciplinary experts: epidemiology, health policy, infectious disease, health services research, implementation science Community-based organization leadership	Strategic visioning and direction Scientific oversight of research prioritization and grantmaking Alignment with state and federal priorities, initiatives and policies
Data Management and Use Committee	Epidemiology Data informatics UC Health legal Compliance and privacy Data stewardship	DUA, data management, privacy protection, and access processes Requests for new datasets, variables, data export requests Requests for CDPH data use outside the current DUA scope
Modeling Research Priorities Governance Committee	CDPH Modeling team CDPH Infectious Disease team UC modelers and lead investigators	Modeling and advanced analytics research priorities Program operations, including Modeling Consortium network meetings
Evidence to Policy Prioritization Committee	Representatives from state agencies, community-based organizations and private sector Includes health and non-health sectors	Alignment with state programmatic priorities and policies Guidance on evidence generation, translation and community engagement activities to improve potential for impact

CDPH, California Department of Public Health; CalHSS, California Health and Human Services; UC, University of California; DUA, Data use agreement.

### COMPONENT 1: Data sharing and evidence generation

One year into the COVID-19 pandemic, in February 2021, CDPH and UC Health launched the UC Health & CDPH Modeling and Advanced Analytics Consortium (hereafter, referred to as the Modeling Consortium), a forerunner to CPR3. The primary goal of this endeavor was to ensure public health policy makers had timely, relevant analyses and insights to support pandemic-related decision-making. With oversight by a Data Management and Use Committee (Table 1), which comprised individuals with operational expertise in public health data sharing, security, and privacy, a core component of this work was ensuring that academic modelers had access to real-time public health surveillance data as the pandemic unfolded.

In March 2021, the Modeling Consortium developed and executed the first ever UC-CDPH wide interagency Data Use Agreement (DUA) that provides a framework for the sharing of COVID-19 surveillance data from CDPH to UC researchers, beyond what is publicly available. This state-wide COVID-19 data includes, for example, laboratory data, deaths, and vaccination rates across state facilities and geographic locations. A novel data platform and process for sharing, cataloging, and hosting the COVID-19 data repository was constructed on UC Research Analysis Environment (UCRAE). UCRAE meets privacy and security requirements, as outlined by the state Information Practices Act (26). For UC researchers to gain access to data in UCRAE, standard operating procedures related to privacy and security safeguards were established, including attestations and renewal processes.

To date, over 60 researchers from seven of the 10 UCs have accessed this data repository. Several studies leveraged these data to generate relevant and useful findings, such as insights into neighborhood-level disparities in COVID-19 cases and vaccination rates, and transmission in K-12 schools (27–29). This platform has become a dynamic entity, now growing to include additional data streams under an expanded CDPH-UC DUA to facilitate data sharing relevant to public health priorities beyond COVID-19. This model has provided a roadmap for how to effectively structure, streamline, and oversee data sharing across state entities. The model supports applied research related to public health emergency response and has become an essential component of pandemic preparedness and health security.

In addition to setting up the DUA for streamlined data sharing, a Rapid Response Consultation and Decision Intelligence Team was created as a component of the Modeling Consortium. CDPH leaders, in consultation with the Modeling Consortium’s Modeling Research Priorities Governance Committee (Table 1), identified eight data modeling experts, representing four of 10 UC campuses, who provided rapid analytic consultation support to CDPH while an in-house CDPH Modeling Team was being built. Analytic support to generate real-time evidence for COVID-19-related policy questions included, for example, short-term forecasts related to case rates, hospitalizations, and ICU occupancy metrics; scenario models assessing the impact of potential public health decisions and behavior change; and health equity metrics of COVID-19 disparities throughout the state of California.

Anchored in monthly touchpoints between the UC consultation team and the CDPH Modeling Team, continuous availability for consultancy has supported both enhanced modeling capacity and infrastructure at the state level and timely data generation through direct data for public health decision-making, separate from publications. The Modeling Consortium influenced several COVID-19 policies including vaccine allocation strategies (23, 30), hospital capacity planning during surges, recommendations for non-pharmaceutical interventions (31), and school openings (27). Analytical outputs also informed projections used on CDPH’s public-facing modeling hub, the California COVID Assessment Tool (CalCAT) which aggregated and vetted a range of models and produced a synthesis of model projections (32). As the need for rapid analysis and modeling related to COVID-19 has waned, this UC consultation mechanism has transitioned into a capacity building team working in support of CDPH’s in-house modeling team for future surges and/or other infectious diseases that impact the state, as well as setting up the infrastructure for more deliberate research and grantmaking activities, such as research agenda setting.

### COMPONENT 2: Shared research agenda setting and implementation

To support targeted decision-making, a Modeling Research Priorities Governance Committee (Table 1) was developed to identify and prioritize key areas of modeling-based research needed by policymakers to gain a deeper understanding of COVID-19 transmission dynamics, impacts of vaccination and non-pharmaceutical interventions, and social and behavioral considerations. In March 2022, four UC research teams were funded through a competitive UC-wide request for proposal (RFP) process to conduct COVID-19-related data analysis and modeling studies (Table 2). In addition to work-in-progress presentations, policy memos, and scientific manuscripts, findings from these modeling studies were shared with key public health leaders and policymakers from CDPH and CalHHS at regular fora (see Component 3). For example, a study led by UC Berkeley used 2020–2023 data on all pediatric cases of COVID-19 reported to CDPH to simulate transmission in schools under different policies; the team uncovered factors likely to be associated with COVID-19 incidence in schools, as well as cases and hospitalizations averted by pediatric vaccinations. These data were presented to the UC-CDPH Modeling Consortium and published (27).

**Table 2:** Research priorities identified and funded through CPR3.

Research priority area	Public health scope	# awards	RFP released
Data-driven modeling to better understand COVID-19 ascertainment, transmission and mitigation strategies, incorporating social and behavioral considerations	Modeling and advanced analytics	4	2021
Policy-relevant modeling to build on lessons learned from the COVID-19 pandemic		3	2024
Impact of the pandemic on children and adolescents and the role of intersecting social, structural and economic factors	Pandemic recovery and readiness	8	2022
Impact of the pandemic on mental health, particularly among sub-populations at increased risk		8	
Impact of the pandemic on social and economic outcomes and evaluation of policies/interventions designed to mitigate or stabilize these effects		8	
Behavior change strategies and public health communication to improve relevance, acceptance and uptake of pandemic-related recommendations, policies and interventions		7	

The success of this component of the Modeling Consortium led to the programmatic expansion of the partnership to better tap into the UC system as a research think tank. The initial focus of CPR3, a research initiative funded by CDPH in July 2022 and administratively housed at UC San Francisco (UCSF), was to better understand the long-term effects of the COVID-19 pandemic on individuals and communities. This grantmaking program was tasked with accelerating public health research to inform recovery and resiliency efforts across California, utilizing the collective strengths of the entire UC system and re-affirming the focus on equity-centered and community-engaged research. To achieve this goal, CPR3 expanded its governance structure to include an Executive Steering Committee (Table 1), enabling the development of a pandemic-focused public health research agenda; created an infrastructure to vet and support high quality research projects that fill policy- and community-relevant evidence gaps; and developed efficient evidence to policy translation processes.

Following scoping activities to synthesize existing evidence and define gaps in recovery-related research, CPR3 established consensus priority areas with public health policymakers to align research goals with policy targets. Along with input from CPR3’s Executive Steering Committee, four focus areas were identified and agreed upon (Table 2). Two topics focused on the impact of the pandemic and its response efforts on the well-being of children and adolescents and on mental health, which align with CalHHS’ Children and Youth Behavioral Health Initiative (CYBHI) and Governor Newsom’s legislation modernizing the Mental Health Services Act (MHSA) (33, 34). CPR3 then developed four RFPs (one for each topic area); RFPs provided details on public health and policy needs within each area, proposal requirements, and information on expectations regarding community engagement, health equity, and collaboration. Before dissemination across the 10 UC schools, each RFP was reviewed by members of the Executive Steering Committee and at least one advisor from a relevant community-based organization.

The CPR3 team implemented an RFP review process that ensured technical rigor, integration of community perspectives, and policy-level feedback on potential for impact and alignment with existing efforts. Specifically, each proposal was evaluated by at least two technical reviewers with methodological/subject matter expertise (including public health experts and clinician-researchers) and one reviewer from a community-based organization. Community engagement and equity was one of the core scoring criteria in addition to research strategy and the investigative team. Priority was given to research that addressed the experiences and needs of individuals and communities who have been disproportionately impacted by the pandemic, as well as teams with strong community or multi-sectoral collaborations. Based on funding recommendations put forth by these review committees, representatives from community-based organizations, policy advisors from relevant states agencies, CalHHS and CDPH, were convened to guide the final investment portfolio. By setting expectations regarding community engagement in RFPs and including community members, clinicians, research experts, and individuals from state agencies in the full process, CPR3 implemented a collaborative grant-making approach focused on health equity using community-driven or community-engaged approaches. Further, within RFPs, CPR3 used an inclusive definition for “research partners,” encompassing community members and creating space for them to serve as co-investigators and full partners in the research process.

The result of these RFP activities includes a portfolio of 31 projects (Table 2) from a range of disciplines, such as legal studies, behavioral and communication sciences, clinical care, implementation science, and more. They include collaborations with over 45 organizations—from community-based organizations to government agencies to school districts which are all critical to the design, execution, interpretation, and dissemination of the projects. Furthermore, projects represent diverse populations, communities, and geographies throughout California, from those living in densely populated urban settings like Los Angeles and the San Francisco Bay Area to those living in rural and agricultural regions, like Yolo, Placer, and Kern Counties. A full list of CPR3-funded studies is available on the CPR3 website with individual project findings published or forthcoming (35–39).

### COMPONENT 3: Building networks for enhanced evidence translation

A critical barrier to translating public health research into practice and policy has historically been the lack of consistent engagement between researchers and public health policymakers in a manner directed at information sharing and problem-solving. To address this challenge, over 30 Modeling Consortium network meetings were held between 2020 and 2023 to discuss high-priority pandemic topics. These virtual meetings brought together CDPH modelers, epidemiologists and decision-makers and over 200 UC faculty with expertise in epidemiology, infectious diseases, economics, statistics, computer science and ecology. While most topics related directly to SARS-CoV-2 (e.g., vaccine effectiveness for new virus variants, wastewater surveillance, evidence for masking, contact tracing, school and university-based transmission, sub-county spatio-temporal modeling, modeling new variants, vaccinations), topics related to other aspects of public health such as health equity and economic impact were also integrated. Since its inception, the scope of these meetings has expanded beyond COVID-19 to include other emerging and endemic infectious diseases affecting Californians, such as RSV disease, mpox, influenza and congenital syphilis. Guest speakers outside of the UC-CDPH network have included individuals from the Centers for Disease Control and Prevention (CDC), the National Institutes of Health (NIH), other academic institutions, state and local public health agencies. This inclusive academic-public health forum has catalyzed transdisciplinary collaboration and promoted applied research to inform state needs.

In addition, while the early phases of the Modeling Consortium relied on CDPH/CalHHS-engaged community advisors, CPR3 aimed to further de-silo academic, community, and policy perspectives by creating the Evidence to Policy Prioritization Governance Committee (Table 1). This committee includes representatives from community-based organizations and state agencies, including the CDPH Office of Health Equity, First 5 California, and the California Black Health Network. It provides input on evidence translation and community engagement activities for CPR3 grantees and helps to ensure alignment with state and policy programmatic priorities and initiatives. Committee members participated in works-in-progress sessions for CPR3-funded projects, whereby they provided critical input on key discussion questions raised directly by the awardees—for instance, around collaboration best practices and how to effectively communicate findings to both policy and advocacy audiences. They also helped awardees identify secondary areas of research, highlight additional measures/outcomes of interest to policymakers, and make connections to relevant individuals or groups. These works-in-progress sessions which concluded in mid-2024 embraced a “show and help” approach for bidirectional learning and networking.

As CPR3-funded studies now transition to dissemination efforts, CPR3 is actively creating shared resources for the broader CPR3 network to enhance evidence translation beyond scientific publishing. Resources include how-to guides for visual abstracts and policy briefs, seminars related to how evidence is used in policy-making, interactive events focused on skill-building for effective research communication, and on-demand support for evidence translation. This support includes helping research teams better understand policy, program-based, and community audiences, their motivations and needs, and how to use insights to translate research findings into recommendations specific to each audience. In Spring 2025, CPR3 hosted an in-person symposium, attended by CPR3-awarded teams and their policy and community partners, CDPH team members and leadership, other state public health and agency officials, and governance committee members. The symposium included keynote speeches, a multi-disciplinary panel, lightning presentations by CPR3 teams, and an evidence translation workshop and roundtable activity.

The CPR3 team also developed a research data catalog—a publicly accessible, searchable website that focuses on public health research datasets in California and links to available open source CDPH data as well (40). The goals of this catalog are: (1) to lower the barriers for individuals to find and access datasets related to COVID-19 and other emerging public health priorities, and (2) to foster inter-disciplinary and inter-institutional collaboration. Through these resources and networking activities, CPR3 is working to foster impactful evidence translation of policy-relevant public health research.

## Discussion

The ongoing partnership between CDPH and the UC system has successfully linked cutting-edge academic research capabilities with the evolving public health needs of California. With oversight provided by core governance structures, CPR3 has enabled the development of shared data sources, provided agile analytic support to public health leaders, and enhanced communication and collaboration among academicians and implementors. As research priorities have evolved from the acute phase of the pandemic to longer-term impacts, community organizations have become a critical partner in this work.

As we continue to strengthen the foundation of CPR3 through this UC-CDPH partnership, three key factors are essential to address for sustainable impact. First, this vision was initially catalyzed by champions from both UC and CDPH with funding provided by the state. However, as many of the federal and state executive actions and programs put in place during the pandemic have since ended, including the availability of dedicated funds for emergency COVID-19 measures and relief funding, identification of sustainable funding to maintain successful infrastructures such as CPR3 is essential so that it is not a “one-off” endeavor. Rather, these types of collaborations should become a core component of public health to support preparedness and response, policy development and community partnerships (41).

Second, sustainability of this multi-pronged partnership requires lowering administrative barriers across government, academic institutions, and community-based organizations. All players are essential to ensure high quality, relevant research is supported across the state, particularly with communities who have been historically under-served or disproportionately impacted by health inequities. This means addressing operational hurdles and streamlining inter-institutional mechanisms by which people can engage in this work. For example, while CPR3 has successfully disbursed funds across all 10 UC campuses, there have been challenges in compensating non-UC individuals for their time, ensuring timely execution of cross-campus contracts, navigating multiple Institutional Review Board requirements, and gaining access to state data that are not part of the above-mentioned DUAs. Another obstacle included navigating state approval processes while adhering to a highly aggressive timeline that far outpaced the timing typically needed to plan, administer funds for, and fully execute research projects.

However, strong leadership and political will embodied in California, its agencies, and the UC system—which was focused on equitably responding to the needs of the state and its people—helped CPR3 create novel solutions to these barriers and develop agile infrastructure to maintain the high levels of scientific rigor and research ethics in timely and pragmatic ways. Focusing on transdisciplinary research and harnessing the multitude of research capabilities throughout the UC system also helped promote innovation and cooperation across campuses. By overcoming some of these institutional administrative barriers, collaboration in research prioritization, implementation and translation can be expedited based on sustaining lessons learned during the pandemic (14).

Lastly, setting research agendas and subsequent investments can be heavily influenced by policy agendas, resource availability, and who has a seat at the table (42). Ongoing collaboration with community organizations is critical for improving how we set priorities, implement actions and policies, and share valuable lessons learned. By maintaining governance structures that are balanced and comprise multiple perspectives, this academic-public health partnership has and will continue to ensure a shared agenda is being set and implemented in California.

We believe this academic-public health infrastructure has vast potential to continue to advance sustainable, high-impact public health research beyond the pandemic by:

(1) Serving as a research agenda development and implementation body that can effectively and efficiently pivot to other important public health topics, such as vector-borne diseases, sexually transmitted diseases, and emerging pandemic threats.
(2) Continuing to improve and streamline data collection, data sharing, and data coordination efforts between public health, health system, and research activities.
(3) Expanding in reach to include other local health jurisdictions and public health agencies, universities, private sector and community-based organizations across the state.

## Conclusion

In this paper, we focus on the establishment of CPR3, a successful academic-public health research partnership to address the COVID-19 emergency and lessons learned. By supporting its vision and including community partners in its reach, the state has created an agile infrastructure that can streamline evidence generation to policy translation and public health implementation.

## Data Availability

The original contributions presented in the study are included in the article/supplementary material, further inquiries can be directed to the corresponding author.

## References

[1] California Department of Public Health. COVID-19 and California’s commitment to health equity. (2024). Available online at: https://www.cdph.ca.gov/Programs/CID/DCDC/Pages/Respiratory-Viruses/Covid-19-Health-Equity.aspx (Accessed on 2024 May 12)

[2] Abrams EM Greenhawt M Shaker M Pinto AD Sinha I Singer A. The COVID-19 pandemic: adverse effects on the social determinants of health in children and families. Ann Allergy Asthma Immunol American College of Allergy, Asthma Immunol. (2022) 128:19–25. doi: 10.1016/j.anai.2021.10.022, PMID: 34699969 PMC8539831

[3] Lyu W Wehby GL. Shelter-in-place orders reduced covid-19 mortality and reduced the rate of growth in hospitalizations. Health Aff. (2020) 39:1615–23. doi: 10.1377/hlthaff.2020.00719, PMID: 32644825

[4] Andrejko KL Pry JM Myers JF Fukui Nozomi Deguzman JL Openshaw J . Morbidity and mortality weekly report effectiveness of face mask or respirator use in indoor public settings for prevention of SARS-CoV-2 infection-California (2021). Available online at: https://www.cdph.ca.gov/Programs/CID/DCDC/Pages/COVID-19/ (Accessed May 12, 2024). 10.15585/mmwr.mm7106e1 PMC8830622 35143470

[5] Sachdev DD Petersen M Havlir DV Schwab J Enanoria WTA Nguyen TQ . San Francisco’s citywide COVID-19 response: strategies to reduce COVID-19 severity and health disparities. Public Health Rep. (2023) 138:747–55. doi: 10.1177/00333549231181353, PMID: 37408322 PMC10323495

[6] Park S Park CG Hong OS. Exploring the characteristics and health outcomes of working from home: analysis of 2021 California health interview survey data. Am J Ind Med. (2024) 67:119–28. doi: 10.1002/ajim.23556, PMID: 38069590

[7] Chaffee BW Cheng J Couch ET Halpern-Felsher B. Engagement, mental health, and substance use under in-person or remote school instruction during the COVID-19 pandemic. J Sch Health. (2024) 94:501–508. doi: 10.1111/josh.13418, PMID: 38086782 PMC11088987

[8] Payán DD Perez-Lua F Goldman-Mellor S Young MEDT. Rural household food insecurity among Latino immigrants during the COVID-19 pandemic. Nutrients. (2022) 14:2772. doi: 10.3390/nu14132772, PMID: 35807952 PMC9268956

[9] Woodward-Lopez G Esaryk E Rauzon S Hewawitharana SC Thompson HR Cordon I . Associations between changes in food acquisition behaviors, dietary intake, and bodyweight during the COVID-19 pandemic among low-income parents in California. Nutrients. (2023) 15:4618. doi: 10.3390/nu15214618, PMID: 37960270 PMC10648135

[10] Grieve R Yang Y Abbott S Babu GR Bhattacharyya M Dean N . The importance of investing in data, models, experiments, team science, and public trust to help policymakers prepare for the next pandemic. PLOS Global Public Health. (2023) 3:e0002601. doi: 10.1371/journal.pgph.0002601, PMID: 38032861 PMC10688710

[11] Turner T El-Jardali F. The crucible of COVID-19: what the pandemic is teaching us about health research systems. Health Res Policy Syst. (2020) 18:52. doi: 10.1186/s12961-020-00573-1 PMC7266130 32487103

[12] Clark J. How covid-19 bolstered an already perverse publishing system. BMJ. (2023) 380:689. doi: 10.1136/bmj.p689, PMID: 36977517

[13] Shea S Nguyen T Kim DH Gee GC Wang MC Umemoto K. Lessons learned from TranslateCovid, a multilingual online resource hub for Asian American and Pacific islander communities and beyond. Public Health Rep. (2024) 139:647–53. doi: 10.1177/00333549241236092, PMID: 38584484 PMC11528835

[14] Casillas A Rosas LG Carson SL Orechwa A North G AuYoung M . STOP COVID-19 CA: Community engagement to address the disparate impacts of the COVID-19 pandemic in California. Front Health Serv. (2022) 2:935297. doi: 10.3389/frhs.2022.935297 PMC10012632 36925779

[15] Chamie G Marquez C Crawford E Peng J Petersen M Schwab D . Community transmission of severe acute respiratory syndrome coronavirus 2 disproportionately affects the latinx population during shelter-in-place in San Francisco. Clin Infect Dis. (2021) 73:S127–35. doi: 10.1093/cid/ciaa1234 32821935 PMC7499499

[16] AuYoung M Rodriguez Espinosa P Chen W. Ting, Juturu P, Young MEDT, Casillas a, et al. addressing racial/ethnic inequities in vaccine hesitancy and uptake: lessons learned from the California alliance against COVID-19. J Behav Med. (2023) 46:153–66. doi: 10.1007/s10865-022-00284-8, PMID: 35066696 PMC8783654

[17] Marquez C Kerkhoff AD Naso J Contreras MG Diaz EC Rojas S . A multi-component, community-based strategy to facilitate COVID-19 vaccine uptake among Latinx populations: from theory to practice. PLoS One. (2021) 16:e0257111. doi: 10.1371/journal.pone.0257111 PMC8452046 34543291

[18] Kerkhoff AD Sachdev D Mizany S Rojas S Gandhi M Peng J . Evaluation of a novel community-based COVID-19 “test-to-care” model for low-income populations. PLoS One. (2020) 15:e0239400. doi: 10.1371/journal.pone.0239400 PMC7546468 33035216

[19] O’Bryan SE Muñoz F Smith D Bearse A Melendrez B Kamdar B . Community based participatory research as a promising practice for addressing vaccine hesitancy, rebuilding trust and addressing health disparities among racial and ethnic minority communities. Hum Vaccin Immunother. (2024) 20:2326781. doi: 10.1080/21645515.2024.2326781, PMID: 38497273 PMC10950264

[20] California Department of Public Health. Blueprint for a safer economy. (2021). Available online at: https://www.cdph.ca.gov/Programs/CID/DCDC/Pages/COVID-19/COVID19CountyMonitoringOverview.aspx (Accessed on 2024 May 1)

[21] California Department of Public Health. COVID-19 and Reopening in-Person Instruction Framework & Public Health Guidance for K-12 schools in California, 2020-2021 school year. (2021). Available online at: https://www.cdph.ca.gov/Programs/CID/DCDC/Pages/COVID-19/COVID19-K12-Schools-InPerson-Instruction.aspx#In-Person%20School%20Reopening (Accessed on May 1, 2024)

[22] California Department of Public Health. Regional stay at home order. (2020). Available online at: https://www.cdph.ca.gov/Programs/CID/DCDC/Pages/COVID-19/Regional-Stay-at-Home-Order-.aspx (Accessed on 2024 May 1)

[23] Hoover CM Estus E Kwan A Raymond K Sreedharan T León T . California’s COVID-19 vaccine equity policy: cases, hospitalizations, and deaths averted in affected communities. Health Aff. (2024) 43:632–40. doi: 10.1377/hlthaff.2023.01163 38709962

[24] University of California. UC facts at a glance. (2023). Available online at: https://ucop.edu/institutional-research-academic-planning/_files/uc-facts-at-a-glance.pdf (Accessed on 2024 Jan 8)

[25] CDPH. CDPH website. (2025). Available online at: https://www.cdph.ca.gov/Pages/About.aspx (Accessed on 2024 Jan 8)

[26] State of California Franchise Tax Board. Information practices act of 1977 (IPA). (2024). Available online at: https://www.ftb.ca.gov/your-rights/privacy/information-practices-act-of-1977.html#:~:text=The%20Act%20requires%20state%20agencies,to%20the%20greatest%20extent%20practicable (Accessed on 2024 Jan 8)

[27] Head JR Andrejko KL Remais JV. Model-based assessment of SARS-CoV-2 Delta variant transmission dynamics within partially vaccinated K-12 school populations. Lancet Reg Health Am. (2022) 5:100133. doi: 10.1016/j.lana.2021.100133 PMC8614621 34849504

[28] Oh DL Kemper KE Meltzer D Canchola AJ Bibbins-Domingo K Lyles CR. Neighborhood-level COVID vaccination and booster disparities: a population-level analysis across California. SSM Popul Health. (2023) 22:101366. doi: 10.1016/j.ssmph.2023.101366, PMID: 36873265 PMC9982676

[29] Oh DL Meltzer D Wang K Canchola AJ DeRouen MC McDaniels-Davidson C . Neighborhood factors associated with COVID-19 cases in California. J Racial Ethn Health Disparities. (2023) 10:2653–62. doi: 10.1007/s40615-022-01443-y, PMID: 36376642 PMC9662780

[30] Chapman LAC Shukla P Rodríguez-Barraquer I Shete PB León TM Bibbins-Domingo K . Risk factor targeting for vaccine prioritization during the COVID-19 pandemic. Sci Rep. (2022) 12:3055. doi: 10.1038/s41598-022-06971-5, PMID: 35197495 PMC8866501

[31] León TM Vargo J Pan ES Jain S Shete PB. Nonpharmaceutical interventions remain essential to reducing coronavirus disease 2019 burden even in a well-vaccinated society: a modeling study. Open forum. Infect Dis Ther. (2021) 8:ofab415. doi: 10.1093/ofid/ofab415, PMID: 34514021 PMC8419741

[32] White LA McCorvie R Crow D Jain S León TM. Assessing the accuracy of California county level COVID-19 hospitalization forecasts to inform public policy decision making. BMC Public Health. (2023) 23:782. doi: 10.1186/s12889-023-15649-0, PMID: 37118796 PMC10141909

[33] CalHHS. CalHHS behavioral health roadmap. (2023). Available online at: https://www.chhs.ca.gov/wp-content/uploads/2023/03/CalHHS-Behavioral-Health-Roadmap-_-ADA-03.02.23.pdf (Accessed May 1, 2024).

[34] CalHHS. Children and Youth Behavioral Health Initiative (CYBHI). (2025). Available online at: https://cybhi.chhs.ca.gov/ (Accessed May 1, 2024).

[35] Raney JH Weinstein S Ganson KT Testa A Jackson DB Pantell M . Mental well-being among adversity-exposed adolescents during the COVID-19 pandemic. JAMA Netw Open. (2024) 7:E242076. doi: 10.1001/jamanetworkopen.2024.2076, PMID: 38477919 PMC10938185

[36] UCSF. California Collaborative for Public Health Research. (2025). Available online at: https://cpr3.ucsf.edu/ (Accessed on 2024 Jun 12)

[37] Maxwell SL McCulloch CE Fernandez A Beck AL. Changes in BMI prior to and during the COVID-19 pandemic among children: a retrospective cohort study in San Francisco, CA. BMC Public Health. (2024) 24:2962. doi: 10.1186/s12889-024-20311-4 PMC11515388 39455995

[38] Raney JH Weinstein S Testa A Ganson KT Memon Z Glidden DV . Sexual identity is associated with adverse childhood experiences in US early adolescents. Acad Pediatr. (2025). 25:102555. doi: 10.1016/j.acap.2024.07.022, PMID: 39134208 PMC11805669

[39] Matias S. Child and adult care food program meal reimbursement rates and program participation by family child care homes in California. J Nutr Educ Behav. (2024) 56:S25–26. doi: 10.1016/j.jneb.2024.05.064 41651590

[40] California Collaborative for Public Health Research. CPR3 Data Catalog. (2024) Available online at: https://data-catalog.cpr3.ucsf.edu/cpr3/s/ (Accessed on 2024 Sep 8)

[41] DeSalvo K Hughes B Bassett M Benjamin G Fraser M Galea S . Public health COVID-19 impact assessment: lessons learned and compelling needs. NAM Perspect. (2021). 2021:10.31478/202104c. doi: 10.31478/202104c PMC8406505 34532688

[42] Meyer JL Waterman C Coleman GA Strambler MJ. Whose agenda is it? Navigating the politics of setting the research agenda in education research-practice partnerships. Educ Policy. (2023) 37:122–46. doi: 10.1177/08959048221131567

